# Neural Mechanisms of Visual–Spatial Judgment Behavior under Visual and Auditory Constraints: Evidence from an Electroencephalograph during Handgun Shooting

**DOI:** 10.3390/brainsci13121702

**Published:** 2023-12-10

**Authors:** Qidi Shi, Anmin Gong, Peng Ding, Fan Wang, Yunfa Fu

**Affiliations:** 1 Faculty of Information Engineering and Automation, Kunming University of Science and Technology, Kunming 650500, China; shiqd2021@163.com (Q.S.); auasr_schorr@163.com (P.D.); wf17853537709@163.com (F.W.); 2 Brain Cognition and Brain-Computer Intelligence Integration Group, Kunming University of Science and Technology, Kunming 650500, China; 3 School of Information Engineering, Chinese People’s Armed Police Force Engineering University, Xi’an 710086, China; 4 School of Life Science and Technology, Xi’an Jiaotong University, Xi’an 710049, China

**Keywords:** shooting, audiovisual limitations, shooting preparation phase, EEG relative band power, ERD/ERS

## Abstract

Light and noise are important factors affecting shooting performance, and shooters can exhibit physiological processes that differ from normal shooting when they are subjected to disturbed visual and auditory conditions. The purpose of this study was to explore the neural mechanism of shooting preparation in skilled shooters with visual and auditory limitations. We designed an experiment and recorded the electroencephalograph (EEG) and shooting performance indexes of 40 individuals skilled in marksmanship during the shooting preparation stage under three conditions: low light, noise interference, and a normal environment. EEG relative band power features and event-related desynchronization/synchronization (ERD/ERS) features were extracted and analyzed. The results showed that (1) the average score of the shooters was 8.55 under normal conditions, 7.71 under visually restricted conditions, and 8.50 under auditorily restricted conditions; (2) the relative EEG band power in the frontal lobe (Fp1, Fp2), frontal lobe (F4, F8), left temporal region (T7), central lobe (CP2), and parietal lobe (P3, PO3) in the theta band was significantly lower than in the other two environments (*p* < 0.05), and there was no significant difference between the power intensity of the shooter in the noisy environment and that in the normal environment; and (3) in the low-light environment, a significant negative correlation was found between the central region, the left and right temporal regions, and the parietal lobe (*p* < 0.05). These findings provide a basis for further understanding neural mechanisms in the brain during the shooting preparation phase under visually and auditorily restricted conditions.

## 1. Introduction

Shooting is a precision sport that is greatly influenced by mental state, and studies have shown that the shooting preparation phase contains complete information about shooting behavior, and neural activity in the brain during the shooting preparation phase directly affects the shooter’s shooting completion level [1]. The analysis of the neural mechanisms of the brain in the shooting preparation stage is a crucial research topic in the field of sports science. Previous studies primarily focused on analyzing the neural mechanisms of the brain in the shooting preparation stage using electroencephalogram (EEG) signals. Therefore, the EEG method has emerged as an essential and classical approach for assessing the neural activity of the brain [2,3]. In the field of sports science, from the perspective of competitive sports and to improve the performance of professional athletes, researchers design experimental paradigms and analyze EEG characteristics to provide effective help for state monitoring, auxiliary evaluation, and psychological training during sports [4,5].

Hatfield et al. (1984) found that the alpha power of the left temporal and occipital regions increased significantly during each shooting preparation, while the activity of the right hemisphere remained constant [6]. Furthermore, Loze et al. compared the alpha band power changes in the occipital region corresponding to the best and worst shots. The results showed an increase in alpha wave power during the best shooting performance and a decrease during the worst shooting performance. Moreover, they suggested that increasing visual attention before shooting is not conducive to achieving good shooting performance [7]. Del Percio et al. found that the amplitude of the ERD of elite athletes in alpha1 (about 8–10 Hz) and alpha2 (about 10–12 Hz) was lower than that of non-athletes. Elite athletes with high-quality shooting scores (about 10–12 Hz) had larger ERS amplitudes for Alpha2 than for low shooting scores [1]. Gallicchio et al. studied the effect of cardiovascular load (e.g., exercise) on shooting performance and the neural mechanisms of the brain during this state and found that a high heart rate load decreased the theta band power of the midfrontal line (Fz, FCz) and increased the alpha band power in the temporal and occipital regions during shooting preparation [8]. Bertollo et al. explored ERD/ERS activity in the theta and alpha bands during shooting preparation. The results suggest that varying levels of motion control also influence EEG signal characteristics during the shooting preparation process [9]. Pereira et al. found that the more asynchronous the mu rhythmic oscillatory activity was during the aiming phase, the better the shooting performance [10]. Gong et al. analyzed the correlations between functional connectivity, brain network topological features, and shooting performance. They identified significant correlations in distinct brain regions within the beta1 and beta2 frequency bands [11]. Gu et al. analyzed and compared EEG brain networks of expert and elite shooters and found that elite shooters had greater functional connectivity in the beta1 and beta2 bands compared to expert shooters [12].

Although many studies have investigated the neural mechanisms of the brain in preparation for shooting, most of these studies were conducted under ideal conditions in a laboratory and did not take into account possible environmental effects in practice. However, in a real environment, light and noise are important factors that may affect shooting, which will have an important impact on shooting performance and brain neural mechanisms in the stage of shooting preparation. Currently, there is a scarcity of studies addressing this topic. At the same time, visual and auditory interference is a common phenomenon and problem in shooting, and visual and auditory interference will have an important impact on shooting behavior. Exploring brain neural mechanisms under conditions of visual and auditory restriction is of great value to revealing relevant brain neural mechanisms and further understanding the brain.

In summary, many scholars have designed different experimental paradigms to study neural mechanisms in the brains of shooters in different states during the preparation phase of shooting. For dynamic analyses of ERD/ERS, EEG power characteristics in different frequency bands, and the correlations between behavioral indicators and EEG power and ERD/ERS in the case of visual and auditory limitations, differences have not been found.

This study hypothesized that (1) an audiovisually restricted condition would have an effect on brain activity during the shooting preparation stage which, in turn, would trigger special neural mechanisms that differ from those in a non-disturbed condition; and (2) there are unique neural EEG markers that are different from those in a normal shooting condition and can reflect shooting behavior indicators. To test these hypotheses, experiments in this study involved the collection of EEG signals from 40 skilled shooters in low-light, noise-interference, and normal environments, as well as seven shooting performance indexes such as the shooting performance, holding stability, and shooting stability of shooters, and analyzed EEG power characteristics and ERD/ERS characteristics to investigate differences in the brain mechanisms of shooters in an audiovisually restricted environment.

## 2. Materials and Methods

### 2.1. Experimental Subject

The subjects were 40 cadets (all male) enrolled at the Armed Police Engineering University, aged 21.2 ± 0.7 years, who had passed a university shooting course and an examination and were proficient in pistol shooting skills. The degree to which the participants could be considered proficient shooters was judged by university experts based on their duration of study and intensity of training [13]. All subjects were physically healthy, free of psychiatric/neurological disorders, and were right-handed. This study stipulated that the subjects should not consume stimulant drinks such as alcohol, coffee, and tea or any neurological drugs that might interfere with the experimental study within 24 h prior to the experiment. Participants participated in the experiment voluntarily, understood the purpose and procedure of the experiment before the experiment, filled out an informed consent form for the experimental protocol, and could report and apply for withdrawal if any discomfort occurred during the experiment. The experiment was approved by the Ethics Committee of the Armed Police Engineering University.

### 2.2. Experimental Environment Setting

The experimental site of the study was located at the indoor shooting training range of the Armed Police Engineering University. Subjects were instructed to shoot in conditions involving noise interference, low light at night, and a normal environment, with the latter characterized by bright lighting and the absence of noise. Before the shooting behavior occurred, the subjects participating in the experiment were required to wear Bluetooth headphones; an irregular shooting sound was played to create the noise interference condition, and the degree of noise interference experienced by the subjects was adjusted using the volume level. In this noisy environment, most of the subjects experienced irritability, difficulty with concentration, and other discomfort, and this volume was the maximum acceptable level of safe noise for most of the subjects. This study used an adjustable incandescent lamp to simulate a low-light environment at night. The criterion for the low-light environment at night was the subject’s difficulty in effectively visually aiming using the level relationship of the collimator gap and the clarity of the target (target paper). The experiment finally chose 25 Lux as the illumination level for the nighttime low-light environment in which which effective visual aiming was not possible.

### 2.3. Experimental Data Acquisition

**Figure 1:**
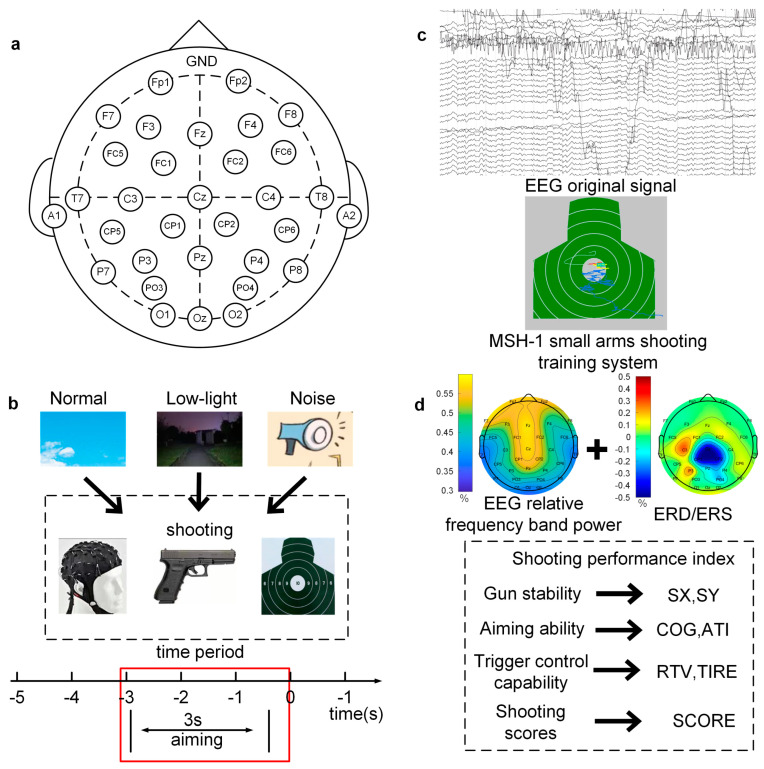
EEG electrode placement and experimental process. (**a**) EEG electrode placement; (**b**) experimental setting process and time interval of aiming stage; (**c**) experimental data acquisition diagram—the upper figure shows EEG data acquisition, and the lower figure shows shooting behavior data acquisition; (**d**) feature extraction and illustration of shooting index.

The EEG acquisition equipment used in this study was a 32-channel wireless EEG amplifier, an NSW332, manufactured by Neuracle Technology Co., Ltd. (Zhejiang, China), which is portable and meets the requirement for mobility in this experiment. The signal sampling rate was 1000 Hz, and 32 EEG acquisition electrodes were placed on the whole scalp according to the international 10–20 standard. As shown in Figure 1a, the electrode positions were as follows: Fp1, Fp2, F7, F3, Fz, F4, F8, FC5, FC1, FC2, FC6, T7, A1, C3, Cz, C4, T8, A2, CP5, CP1, CP2, CP6, P7, P3, Pz, P4, P8, PO3, PO4, O1, Oz, and O2 with forehead grounding; A1 and A2 were reference electrodes which were placed at the left and right mastoid processes, and the average value of the two mastoid processes was taken as a reference. Before the experiment, the electrode impedance was adjusted to below 5 kΩ, and both the resting EEG and shooting EEG were then collected.

First, the EEG signal was collected in the resting state, and the subjects were asked to remain relaxed, to not recall anything intentionally, and to maintain a resting state with eyes closed for 2.5 min and a resting state with eyes open for 2.5 min.

Second, the whole EEG signal of the shooting phase was collected. The shooting target used a standard chest ring target paper chart, and the size of the target was 52 × 52 cm with 10 rings. The diameter of the 10th ring was 10 cm, and the rings’ edges expanded 5 cm outward for rings 9, 8, 7, 6, and 5. The shooting performance was recorded as 5–10 rings according to the target (off-target was recorded as 0 rings). The experiments in this study were conducted using a Type 92 pistol in a standing position, combined with an optical shooting training system, to simulate shooting without live ammunition in three environments: noise interference, low light, and bright and noise-free conditions. In the low-light condition, the subjects were placed in the set low-light environment for about 8 min to allow them to adapt to the low-light environment in advance. All subjects carried out the shooting process independently, and the subjects were not aware of each other’s performances and were instructed prior to the test to not pay too much attention to their performance and instead to focus on their shooting skills. The subjects fired each shot in the experiment, the EEG recorder provided feedback on the shooting performance, and the shooter then adjusted their aiming point according to this performance and proceeded to aim the next shot. The subjects were free to fire at their own pace, and two groups of 30 shots were fired each of the three experimental settings, with each subject firing a total of 180 shots; the interval between each group of shots was ten minutes. The shooting moment was recorded by the Trigger Box, which was used as an EEG signal to mark the shooting point by recognizing the moment at which the sound was emitted during each shot in the shooting training system.

In the experiment, the MSH-1 light weapons shooting training system produced by Beijing Zhongkejiecheng Technology Co., Ltd. (Beijing, China) was used to automatically collect performance data for each shot. The system is depicted in Figure 1c. The system uses the light reflection principle to realize the aiming function of pistol shooting. The computer feedback interface shows the track of the tracing point and can record and store shooting performance data for each shot. After each shot, the system comprehensively evaluates the shooting performance of the shooter from three perspectives, gun, aiming, and trigger, and records seven shooting performance indicators, SX, SY, COG, ATI, RTV, TIRE, and SCORE. The meaning of each shooting index is provided in Figure 1d. The behavior indicator data were 3 × 7 × 60 × 40 (3 environmental conditions, shooting behavior index, trial times, and subjects).

### 2.4. Signal Preprocessing

The original EEG data for the three conditions were collected in 32 × 5000 × 60 × 40 format, with each dimension representing 32 channels, among which the 17th and 18th channels were reference electrodes (A1 and A2), 5000 data sampling points (5 s data before shooting), 60 trials, and 40 subjects. The EEG signals were transmitted to MATLAB for off-line analysis and processing. First, a finite impulse response (FIR) of the order of 200 was used to conduct bandpass filtering at 0.1–50 Hz for the data signals. Second, the filtered signals were segmented, and the shooting moment was recorded as 0 s; the EEG signals from 3 s before the shooting moment to the shooting moment were intercepted and recorded as a trial. The time window segmentation is illustrated in Figure 1b, and periods of −3–−2 s, −2–−1 s, and −1–0 s were recorded as time windows Win1, Win2, and Win3. The segmented signals were divided into frequency bands. To reduce individual differences among the experimental subjects, this study adopted a method using the individual alpha frequency (IAF) to determine the frequency divisions of different subjects. The IAF refers to the peak frequency of a subject’s brain at 8~12 Hz [14]. The Fast Fourier Transform (FFT) method was used to calculate the power of the occipital region channels (O1 and O2) in the range of 8~12 Hz in the resting state with eyes closed. The frequency position of the power in the highest frequency band was taken as the IAF of the subject. Then, according to the IAF, the sub-band frequency of each subject was determined: the theta band, alpha band, and beta band were defined as IAF-6-IAF-3 (Hz), IAF-2-IAF+2 (Hz), and IAF+3-IAF+20 (Hz), respectively. After that, an Independent Component Analysis (ICA) was performed on the data of each subject to remove EOG artifacts.

### 2.5. EEG Power Dynamic Analysis

In this study, the Individual Alpha Frequency (IAF) method was used to determine the frequency bands of different subjects to exclude individual differences as much as possible [15]. According to the theta, alpha, and beta bands divided by the IAF of each subject, corresponding FIR bandpass filters were designed to filter the EEG signals of all subjects, and filtered EEG signals were obtained in specific frequency bands. The relative band power method was used to study dynamic changes in the EEG power of the shooters in different environments. The relative band power of the filtered EEG signals was calculated by dividing the signal power of the frequency band by the EEG power of the whole band (4–30 Hz) to obtain the relative band power of the frequency band [16], and the calculated EEG relative frequency band power was divided into frequency bands to dynamically analyze the change trend according to the change in the time window. The calculation formula is as follows:

(1) $$P_{Relation\;frequency}=\frac{P_{frequency}}{P_{theta}+P_{alpha}+P_{beta}}$$

where $P_{frequecy}$ is the EEG power value of a certain frequency band, $P_{Relative\;frequency}$ is the EEG relative frequency band power of this frequency band, and $P_{theta}$, $P_{alpha},$ and $P_{beta}$ represent the EEG power values of the theta band, alpha band, and beta band, respectively. At the same time, the filtered EEG signals were squared, and a logarithmic operation was performed to convert the EEG power into decibels (dB) so as to obtain the absolute EEG power of the subjects in different frequency bands in the three environments. In order to remove obviously abnormal interference data, outliers were removed from the calculated power data to obtain the relative frequency power of the theta, alpha, and beta bands and the EEG power in the form of decibels (dB). The EEG data format of the subjects in noise-interference, low light at night, and normal environments was 32 × 3 × 4 × 40. Each dimension represents 32 channels, 3 frequency bands, 4 time windows (the fourth time window is 1 s after the shooting moment, namely 0–1 s), and 40 subjects, respectively.

### 2.6. Characteristics Analysis of ERD/ERS

In order to quantify the changes in event-related EEG power, we calculated the ERD/ERS characteristics of the subjects in the theta, alpha, and beta bands under three conditions [17,18]; the formula is as follows:

(2) $$ERD/ERS=\frac{P_{event}-P_{baseline}}{P_{baseline}}\times 100\%$$

The EEG signals were divided into four time windows (−4–−3 s, −3–−2 s, −2–−1 s, and −1–0 s). The EEG signal power of each window was calculated and represented as the EEG power characteristic of this window. The power characteristics of the four time Windows obtained are denoted as T1, T2, T3, and T4. The baseline state is −4–3 s which, according to previous studies, is generally considered the resting state when the target is about to start [19,20,21]. $P_{event}$ is the EEG power value of a time window;$\;P_{beseline}$ is the EEG power value at baseline (−4–−3 s).

### 2.7. Statistical Analysis

The Kolmogorov–Smirnov test revealed that the experimental samples all followed a Gaussian distribution. The test results indicated that all the data conformed to a normal distribution (*p* > 0.05). Therefore, the primary test used was a repeated-measures ANOVA.

In the processing of behavior indicator data, 7 shooting indicators of 40 subjects in three conditions were obtained by averaging the trial dimensions. The 7 behavioral indicators of the three conditions were respectively analyzed using a one-way analysis of variance (condition factor).

It was hypothesized that EEG relative band power and time window characteristics are significantly different from normal ones under noise-interference and low light conditions. To test for differences, the EEG power was analyzed using a two-factor analysis of variance (condition factor and time window factor).

Similar to the statistical analysis of EEG relative band power characteristics, this study aims to explore differences in the ERD/ERS characteristics of shooters in different environments. For ERD/ERS characteristics in different environments, the two-way RM ANOVA analysis method was used to study the significant differences in ERD/ERS characteristics in each lead in the theta, alpha, and beta frequency bands, considering both condition factors and time window factors (Win1, Win2, and Win3).

In order to reveal the association between shooting performance and EEG characteristics under different conditions, seven shooting behavior indicators and EEG power and ERD/ERS characteristics were analyzed using their Pearson correlation coefficients [22].

The significant levels of the above statistical analyses were all set to 0.05, and the statistical results were set to 0.01 for very significant results. A post hoc method, specifically the Scheffe type, was used for multiple comparisons of the significant results. The previously described procedures utilized the Statistics and Machine Learning Toolbox in MATLAB 2020b.

## 3. Results

### 3.1. Differences in Shooting Behavior Indicators

**Figure 2:**
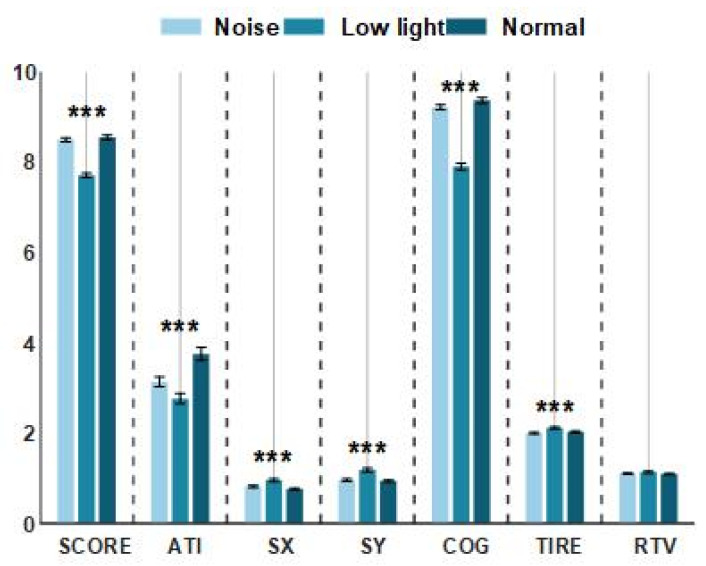
The performance of shooting behavior indexes under normal conditions, low-light conditions, and noise conditions. The data in the figure are the mean values of the shooting performance indicators of the shooters in different environments. The horizontal axis represents the shooting behavior targets, and the vertical axis represents the level of the shooting behavior performance of the shooters. The asterisks (***) in the figure denote significant differences among the normal conditions, low-light conditions, and noise conditions.

As shown in Figure 2, there was no significant difference between the noise conditions and the normal conditions in the three indicators of shooting performance (SCORE), the aiming time (ATI) taken to aim the point on the target, and the mean gravity of the aiming curve (COG), while performance in the low-light condition for these three indicators was significantly lower than for the control condition. However, from the perspective of four indicators, namely, the average deviation value of the horizontal movement of the aiming point (SX), the average deviation value of the vertical movement of the aiming point, the trigger mass (TIRE), and the relative trigger value (ATV), performance in the low-light condition was higher than in the control condition, but there was no significant difference between the noise condition and the control condition. As shown in Table 1, the results of a statistical analysis showed that there was no significant difference in the RTV among the three conditions (*p* > 0.05). There were significant differences in the ATI among the three conditions (*p* < 0.01). The results of the post hoc comparison showed that the control condition was the largest, followed by the noise condition, and the low-light condition was the least. In addition to these two indicators, the results of the other five indicators showed that the three conditions had significant differences (*p* < 0.01). The comparison after the event showed that for the noise condition and the control condition, the subjects exhibited similar shooting behaviors, but the mean values of the SX, SY, and TIRE indexes for the control condition were lower than those for the low-light condition, and the mean values of the SCORE and COG indexes were higher than those in the low-light condition.

### 3.2. Dynamic Difference in Relative EEG Band Power

In this experimental study, significant differences in EEG relative frequency band power characteristics across the condition and time window factors were obtained via a two-way RM ANOVA as follows.

**Figure 3:**
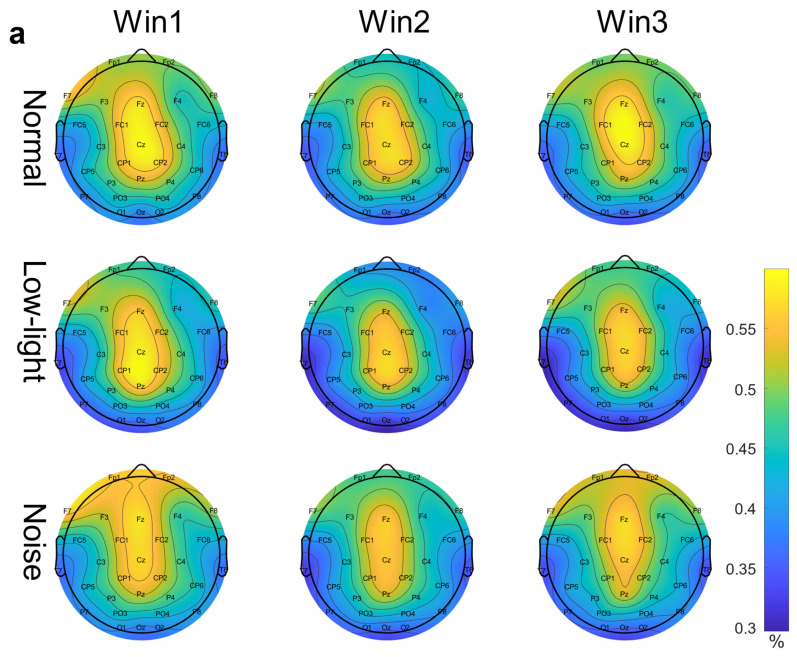

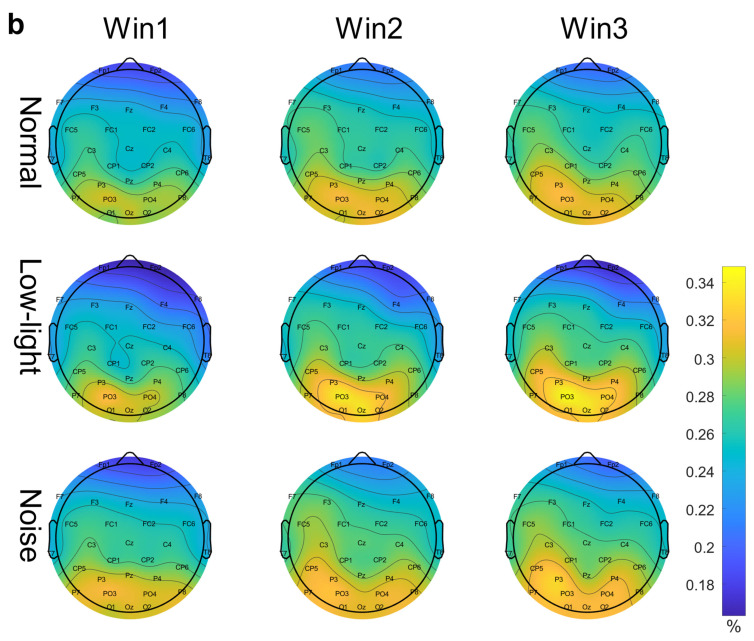

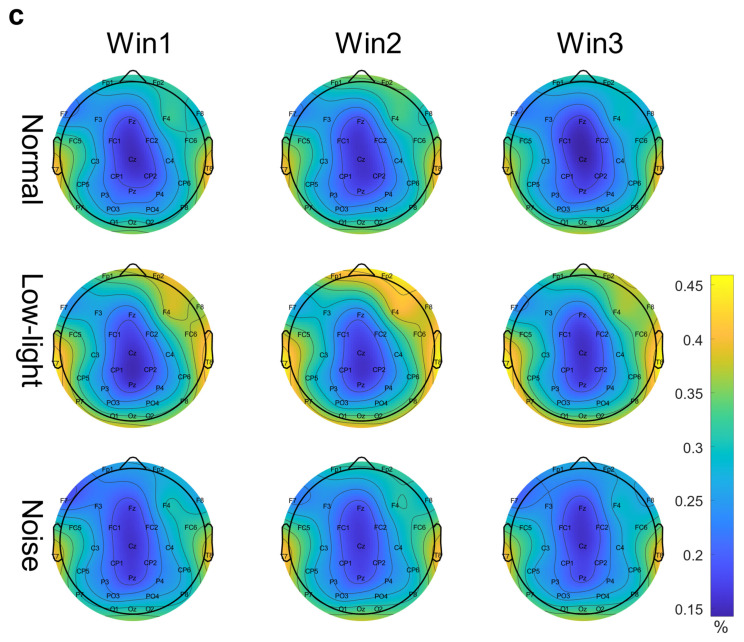
EEG relative band power brain topographic map. (**a**) Theta band EEG relative band power brain topographic map; (**b**) alpha band EEG relative band power brain topographic map; (**c**) beta band EEG relative band power brain topographic map. The figure shows the differences in EEG relative band power characteristics in different time windows (Win1, Win2, and Win3) in the normal condition, low-light condition, and noise condition, where a represents the theta frequency band, b stands for the alpha band, and c stands for the beta band. The data in the figure are the total mean of the scalp topography distribution of EEG relative band power. The color difference in the figure represents the strength of EEG relative band power features. The colder the color, the weaker the EEG relative band power features, and the warmer the color, the stronger the EEG relative band power features.

**Figure 4:**
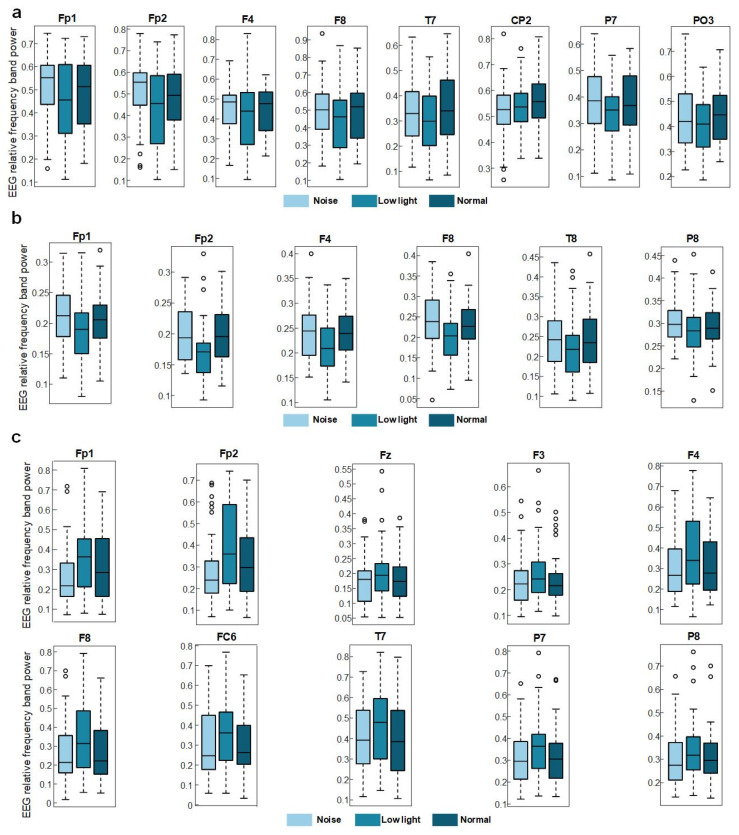
EEG relative band power of shooters in low-light, noisy, and normal environments. (**a**) Significant differences in the theta frequency band. (**b**) Significant differences in the alpha frequency band. (**c**) Significant differences in the beta frequency band. The data in the figure are the EEG relative band power in three conditions. The darker color is the normal condition, the progressively lighter one is the low-light condition, and the lightest one is the noise condition.

Figure 3a shows the differences in EEG relative band power in different time windows of the theta band (Win1, Win2, and Win3) for the normal condition, low-light condition, and noise condition. The figure showed that in the theta band, the relative EEG band power intensity for the normal condition, the noise condition, and the low-light condition was concentrated in the middle and upper left part of the topographic map of the brain, and the power of the noise condition in the prefrontal lobe was stronger than that of the normal condition and the low-light condition. Compared with the normal condition, the power of the left and right temporal and occipital lobes was lower in the low-light condition. In terms of the time window factor, the power intensity of the three conditions in the prefrontal lobe decreased first and then increased with an increase in the time window and was the weakest in Win2. As shown in Figure 4a, the significant differences among the condition factors in the two-way RM ANOVA were reflected in the Fp1 (F = 4.58, *p* < 0.05), Fp2 (F = 7.07, *p* < 0.01), F4 (F = 3.31, *p* < 0.05), F8 (F = 3.91, *p* < 0.05), T7 (F = 3.31, *p* < 0.05), CP2 (F = 4.29, *p* < 0.05), P7 (F = 4.58, *p* < 0.05), and PO3 (F = 3.07, *p* < 0.05) nodes; significant differences among time window factors were observed at the Fp1 (F = 6.05, *p* < 0.01), Fp2 (F = 5.94, *p* < 0.01), F3 (F = 3.52, *p* < 0.05), and F7 (F = 4.47, *p* < 0.05) nodes. The corresponding statistical analysis is presented in Table 2.

Figure 3b shows the differences in the EEG relative band power in different time Windows (Win1, Win2, and Win3) of the alpha band among the normal condition, low-light condition, and noise condition. The figure shows that in the alpha band, the EEG relative band power intensity for the normal, noise, and low-light conditions was concentrated in the lower part of the brain topographic map, and the power in the low-light condition in the parietal and occipital lobes was stronger than that in the normal and noise conditions. Compared with the normal condition, the power of the parietal and occipital lobes in the noisy condition was also stronger than in the normal condition. In terms of the time window factor, the power intensity for the three conditions in the parietal and occipital lobes gradually increased with an increase in the time window and was the strongest in Win3. As shown in Figure 4b, the significant differences among the condition factors of the two-way RM ANOVA were reflected in the Fp1 (F = 6.12, *p* < 0.01), Fp2 (F = 10.9, *p* < 0.01), F4 (F = 11.6, *p* < 0.01), F8 (F = 8.16, *p* < 0.05), T8 (F = 3.03, *p* < 0.05), and P8 (F = 3.99, *p* < 0.05) nodes; significant differences among time window factors were found in Fp1 (F = 7.96, *p* < 0.01), Fp2 (F = 6.78, *p* < 0.01), Fz (F = 3.35, *p* < 0.05), F3 (F = 6.55, *p* < 0.01), F4 (F = 3.14, *p* < 0.05), F7 (F = 4.73, *p* < 0.01), F8 (F = 3.47, *p* < 0.05), FC5 (F = 3.32, *p* < 0.05), C4 (F = 3.14, *p* < 0.05), CP1 (F = 4.85, *p* < 0.01), P4 (F = 3.15, *p* < 0.05), and O2 (F = 3.41, *p* < 0.05). The corresponding statistical analysis is presented in Table 3.

Figure 3c shows the differences in the EEG relative band power in different time windows of the beta band (Win1, Win2, and Win3) among the normal, low-light, and noise conditions. The figure shows that in the beta band, the relative EEG band power intensity for the normal condition, low-light condition, and noise condition was concentrated in the left and right temporal regions and occipital lobe, and the power for the low-light condition was concentrated in the left and right temporal regions and for the prefrontal lobe was stronger than that of the normal and noise conditions. There was no significant difference in EEG power between the noise condition and the normal condition. In terms of the time window factor, the normal condition and the noise condition did not show a change in power with a change in the time window, but in the low-light condition, the prefrontal area and the left frontal area first increased and then decreased with an increase in the time window and the strongest occurred in Win2. As shown in Figure 4c, the significant differences among the condition factors of the two-way RM ANOVA were reflected in the Fp1 (F = 7.94, *p* < 0.01), Fp2 (F = 11.0, *p* < 0.01), Fz (F = 5.71, *p* < 0.01), F3 (F = 3.08, *p* < 0.05), F4 (F = 7.95, *p* < 0.01), F8 (F = 9.64, *p* < 0.01), FC6 (F = 4.46, *p* < 0.05), T7 (F = 3.12, *p* < 0.05), P7 (F = 5.04, *p* < 0.01), and P8 (F = 5.09, *p* < 0.01) nodes; there was no significant difference between the time window factors. The corresponding statistical analysis is presented in Table 4.

### 3.3. Differences in ERD/ERS Characteristics

In this experimental study, significant differences in ERD/ERS characteristics across condition factors and time window factors were obtained via a two-way RM ANOVA as follows.

**Figure 5:**
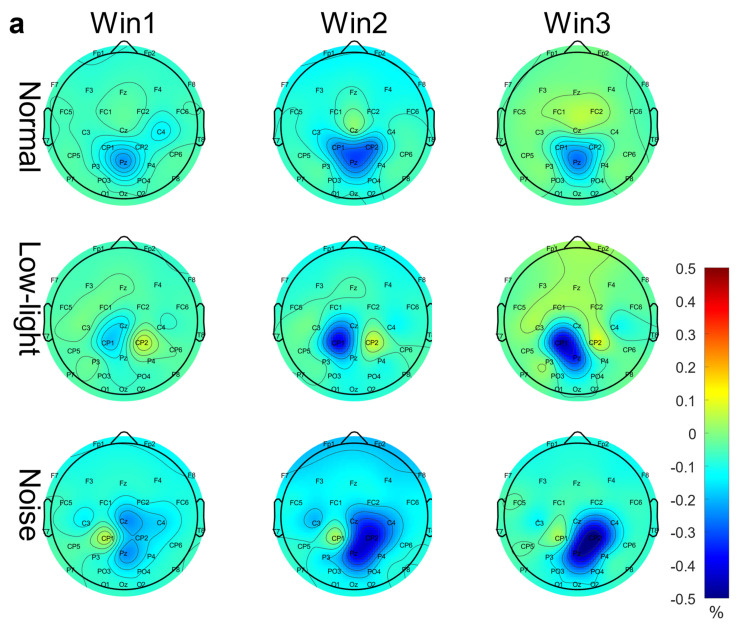

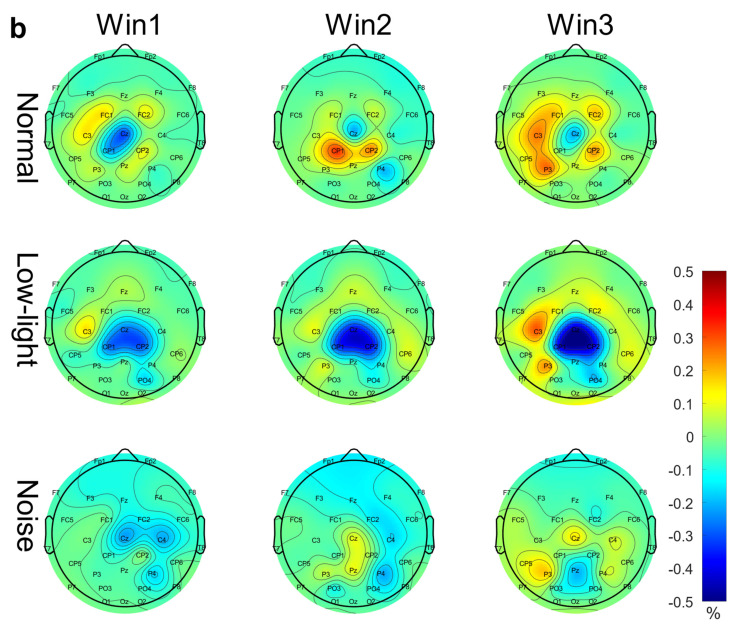

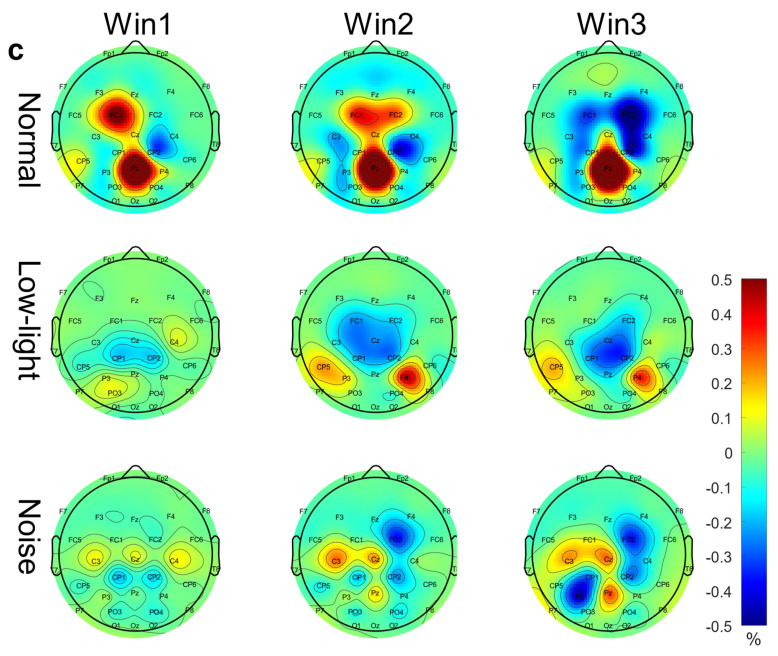
ERD/ERS brain topographic map in low-light, noisy, and normal environments. (**a**) ERD/ERS brain mapping in the theta band. (**b**) ERD/ERS brain mapping in the alpha band. (**c**) ERD/ERS brain mapping in the beta band. The figure shows the differences in ERD/ERS characteristics in different time windows (Win1, Win2, and Win3) in the theta, alpha, and beta bands in the normal condition, low-light condition, and noise condition. The data in the figure are the population average of the scalp terrain distribution of ERD/ERS amplitudes. The color difference in the figure represents the intensity of ERD/ERS features. The colder the color, the stronger the ERD feature, and the warmer the color, the stronger the ERS feature.

As shown in Figure 5a, in the theta band, the ERD characteristics in the normal condition, the noise condition, and the low-light condition gradually increased with an increase in the time window, but the nodes displaying the ERD characteristics in each condition were different, and the ERD characteristics for the normal condition were more obvious at CP1, CP2, and Pz; the ERD characteristics in CP1 and Pz were more obvious in the low-light condition; and for the noise condition, the ERD characteristics in Cz, CP2, and Pz were more obvious. The significant differences between the conditions in the two-way RM ANOVA were reflected in the Fp1 (F = 9.98, *p* < 0.01), Fp2 (F = 8.81, *p* < 0.01), Fz (F = 6.03, *p* < 0.01), F3 (F = 4.66, *p* < 0.05), F4 (F = 4.74, *p* < 0.01), F7 (F = 6.09, *p* < 0.01), F8 (F = 4.13, *p* < 0.05), FC1 (F = 3.49, *p* < 0.05), FC2 (F = 4.57, *p* < 0.05), FC6 (F = 3.17, *p* < 0.05), CP1 (F = 4.09, *p* < 0.05), CP2 (F = 4.06, *p* < 0.05), CP6 (F = 3.60, *p* < 0.05), P4 (F = 3.52, *p* < 0.05), P8 (F = 5.32, *p* < 0.01), PO3 (F = 3.32, *p* < 0.05), PO4 (F = 4.84, *p* < 0.01), O1 (F = 4.06, *p* < 0.05), and O2 (F = 3.81, *p* < 0.05) nodes. Significant differences among time window factors were found in the Fp1 (F = 14.4, *p* < 0.01), Fp2 (F = 16.5, *p* < 0.01), Fz (F = 5.57, *p* < 0.01), F3 (F = 6.89, *p* < 0.01), F4 (F = 7.30, *p* < 0.01), F7 (F = 7.03, *p* < 0.01), F8 (F = 5.97, *p* < 0.01), FC1 (F = 6.95, *p* < 0.01), FC2 (F = 5.62, *p* < 0.01), FC5 (F = 6.28, *p* < 0.01), FC6 (F = 4.15, *p* < 0.05), and O1 (F = 3.62, *p* < 0.05) nodes. The corresponding statistical analysis is presented in Table 5.

As shown in Figure 5b, in the alpha band, for the normal condition as a whole, ERS features were concentrated around the Cz node, and ERS enhanced with an increase in the time window, while the ERD features were weak at Cz. In the low-light condition, Cz, CP1, and CP2 showed strong ERD characteristics, and the ERS characteristics of the surrounding nodes gradually increased with an increase in the time window. Different from the other two conditions, the noise conditions changed from ERD characteristics to ERS characteristics with an increase in the time window at Cz, C4, and P4, while the ERS characteristics gradually increased at P3 and CP5. The significant differences between the conditions in the two-way RM ANOVA were reflected in the Fp1 (F = 7.60, *p* < 0.01), Fp2 (F = 9.35, *p* < 0.01), Fz (F = 8.25, *p* < 0.01), FC1 (F = 5.34, *p* < 0.01), Cz (F = 5.28, *p* < 0.01), C3 (F = 3.31, *p* < 0.05), T8 (F = 3.54, *p* < 0.05), and CP5 (F = 5.18, *p* < 0.01) nodes. Significant differences among time window factors were found in the Fp1 (F = 14.4, *p* < 0.01), Fp2 (F = 16.5, *p* < 0.01), F3 (F = 6.89, *p* < 0.01), F4 (F = 7.30, *p* < 0.01), FC5 (F = 7.03, *p* < 0.01), FC6 (F = 7.03, *p* < 0.01), T7 (F = 7.03, *p* < 0.01), P3 (F = 7.03, *p* < 0.01), P7 (F = 7.03, *p* < 0.01), and P8 (F = 7.03, *p* < 0.01) nodes. The corresponding statistical analysis is presented in Table 6.

As shown in Figure 5c, in the beta band, the normal conditions showed strong ERD characteristics at the Pz node, FC1 and FC2 showed ERS characteristics in Win1 and Win2 but changed to ERD characteristics in Win3 with an increase in the time window. In the low-light condition, ERD characteristics were found in the central region, and the characteristics were enhanced gradually with an increase in the time window, and the ERS characteristics at CP5 and P4 were also enhanced with the time window. The noise conditions have ERS characteristics at C3, C4, and Pz and ERD characteristics at P3, CP1, CP2, and FC2. The ERD/ERS characteristics for the noise condition at each node are enhanced with an increase in time window. The significant differences between the conditions in the two-way RM ANOVA were reflected in the F4 (F = 3.59, *p* < 0.05), F7 (F = 4.41, *p* < 0.05), FC5 (F = 5.76, *p* < 0.01), FC6 (F = 3.52, *p* < 0.05), Cz (F = 7.01, *p* < 0.01), C3 (F = 6.88, *p* < 0.01), T7 (F = 6.78, *p* < 0.01), T8 (F = 3.89, *p* < 0.05), and CP5 (F = 4.22, *p* < 0.05) nodes. Significant differences among time window factors were found in the Fp1 (F = 5.01, *p* < 0.01), Fp2 (F = 7.43, *p* < 0.01), F4 (F = 5.54, *p* < 0.01), P7 (F = 6.01, *p* < 0.01), and O1 (F = 3.65, *p* < 0.05) nodes. The corresponding statistical analysis is presented in Table 7.

### 3.4. Correlation between Shooting Performance and EEG Power Characteristics

#### 3.4.1. Correlation between Shooting Performance and EEG Power Characteristics in Theta Band

**Figure 6:**
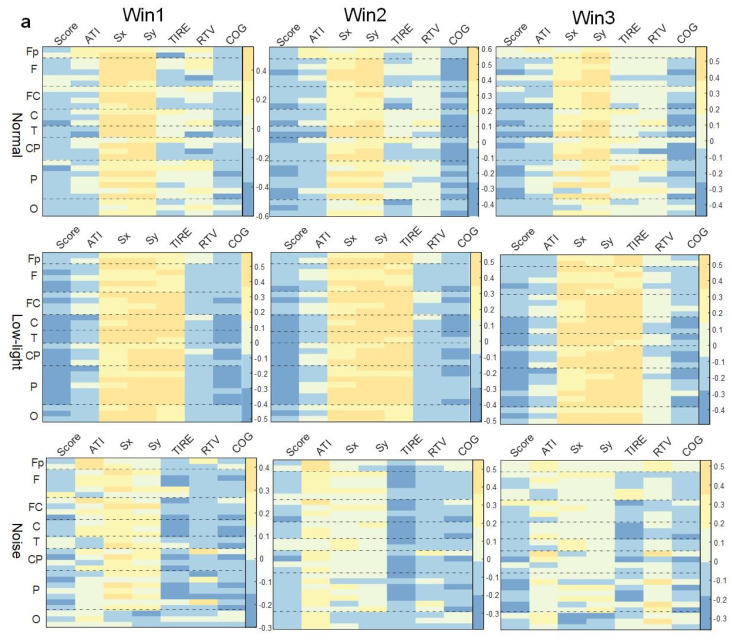

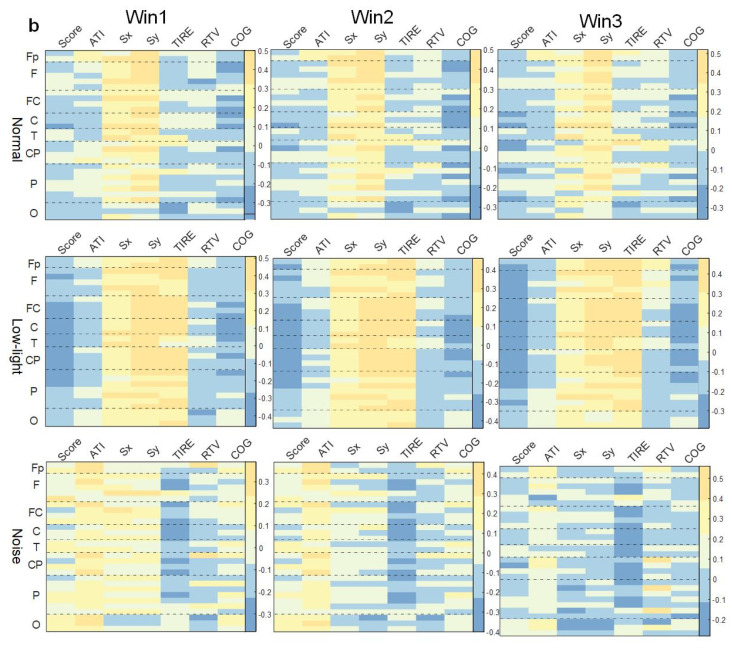

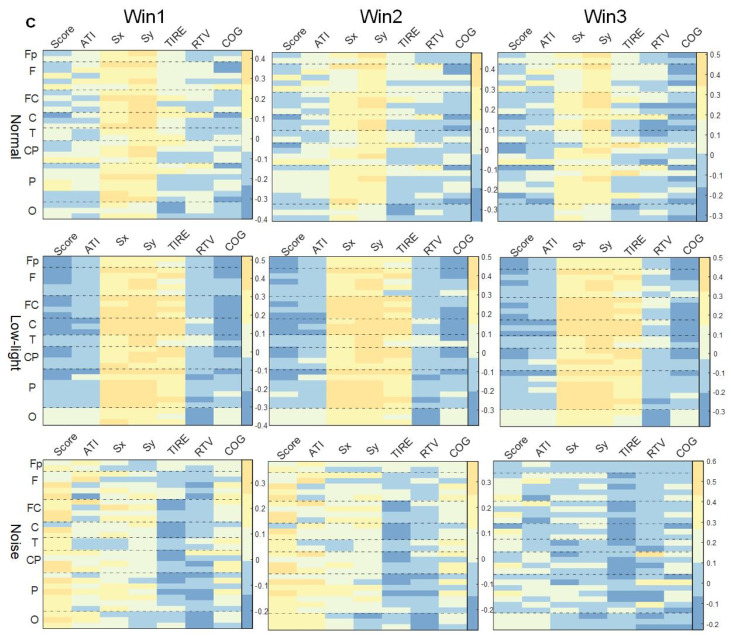
Correlation between EEG relative band power characteristics and shooting performance. (**a**) Theta band correlation; (**b**) alpha band correlation; (**c**) beta band correlation. This figure shows the correlation difference between the EEG relative band power characteristics in the normal, low-light, and noise conditions in different time windows (Win1, Win2, and Win3) of the theta, alpha, and beta bands and the corresponding shooting performance, where (**a**) represents the theta band, (**b**) represents the alpha band, and (**c**) represents the beta band. The data in the figure are Pearson correlation coefficients. The vertical coordinates represent the leads of the corresponding brain regions. The color differences in the graph represent the magnitude of the correlation coefficients; the cooler the color, the smaller the correlation coefficient, and the warmer the color, the larger the correlation coefficient.

As shown in Figure 6a, in the theta band, the correlation between EEG power and behavioral indexes in the low-light condition was stronger than in the normal condition, while the correlation was weakest in the low-light condition, and the correlation did not change significantly with an increase in the time window. The correlation between EEG power and the Sx and Sy indexes in the normal condition, low-light condition, and noise condition was positive at all leads. It was found that the positive correlation between the Sx and Sy indexes in the low-light condition was stronger than in the normal and noise conditions; meanwhile, for the normal condition, it was stronger than for the noise condition. In the normal condition, there was a significant positive correlation between Fp2, Fz, F3, F4, F7, F8, FC2, FC5, C3, C4, T7, T8, CP2, CP5, CP6, P4, P8, PO4, and O2 (*p* < 0.05). The low-light condition showed a significant positive correlation with Fp2, Fz, F7, F8, FC1, FC2, FC5, Cz, C3, C4, T7, T8, CP5, CP6, Pz, P4, P7, P8, PO3, PO4, Oz, O1, and O2 (*p* < 0.05). The noise condition was positively correlated with Fz, FC2, and P8 (*p* < 0.05). In the correlation study of the SCORE index, it was found that the correlations between the normal condition and the noise condition were basically the same but for the low-light condition, the EEG power was more negatively correlated with this index, and all the leads were significantly negatively correlated at F8, FC2, Cz, C3, C4, T7, T8, CP2, CP5, CP6, P3, P4, P7, P8, PO3, PO4, and O1 (*p* < 0.05).

#### 3.4.2. Correlation between Shooting Performance and EEG Power Characteristics in Alpha Band

As shown in Figure 6b, in the SCORE index study, it was found that the negative correlation was stronger in the low-light condition than in the normal condition, and the leads with a negative correlation were concentrated in the frontal lobe, central region, temporal lobe, and parietal lobe; on the contrary, the correlation was relatively weak for the noise condition, and only most of the leads showed a weak positive correlation; the same was true in the normal condition without any significant correlation. Additionally, the correlation between the Sx and Sy indexes and EEG power in this band was similar to that in theta band, and the correlation between the low-light condition and the other two conditions was stronger. The low-light condition showed a dynamic change in correlation with an increase in the time window in this band, but there was no strong correlation before and after the change. In the correlation study with the TIRE index, it was found that the low-light condition presented a positive correlation, and the leads with a significant positive correlation (*p* < 0.05) were Fp1, Fp2, Fz, F3, F4, F8, FC1, FC2, FC5, FC6, Cz, C3, C4, T7, CP1, CP2, CP5, CP6, Pz, P4, PO3, PO4, and O2. The correlations of the noise condition were similar to the normal condition, but the negative correlation was stronger, and the significant negative correlation (*p* < 0.05) was concentrated in the left central region (C3) and the right central region (FC6, C4, CP2).

#### 3.4.3. Correlation between Shooting Performance and EEG Power Characteristics in Beta Band

As shown in Figure 6c, the correlation between EEG power in the beta band and various behavioral indicators also did not change significantly with an increase in the time window. In the study of the SCORE index, there was no significant correlation with the normal condition; in the low-light condition, the prefrontal (Fp2), central frontal (Fz), central (Cz), left central (CP1), right central (FC6, C4, CP2), and central parietal (Pz) lobes were significantly negatively correlated (*p* < 0.05). Contrary to the low-light condition, the noise condition showed significant positive correlations in the right parietal (P4), right frontal (F8), left central (C3, CP5), and left parietal (P3, P7) lobes (*p* < 0.05). In the correlation study of the Sx and Sy indexes, similar to the theta band and alpha band, the low-light condition also showed a strong positive correlation with the other two conditions. The leads with a significant positive correlation were Fz, F4, F7, FC1, FC2, FC5, FC6, Cz, C3, C4, T7, T8, CP1, CP2, CP5, CP6, Pz, P4, P7, P8, PO3, PO4, Oz, and O2. In the normal condition, leads were significantly positively correlated with Fp1, Fz, FC2, FC5, CP5, C3, C4, P4, and PO4. The noise condition did not find a significant correlation with the Sx and Sy index nodes.

### 3.5. Correlation between Shooting Performance and ERD/ERS Characteristics

#### 3.5.1. Correlation between Shooting Performance and ERD/ERS Characteristics in Theta Band

**Figure 7:**
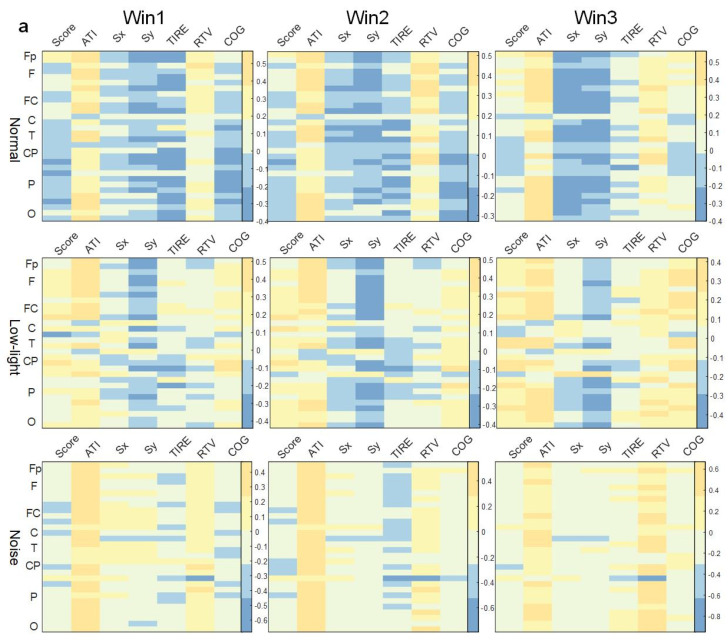

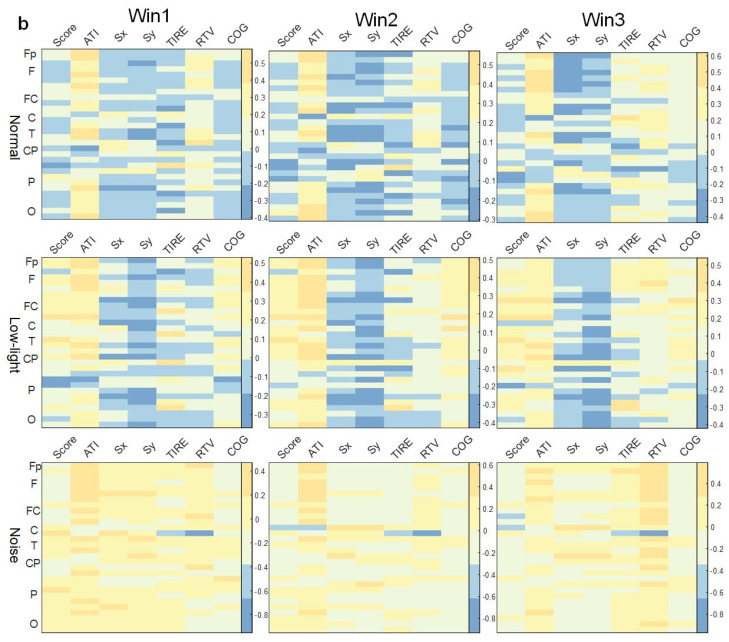

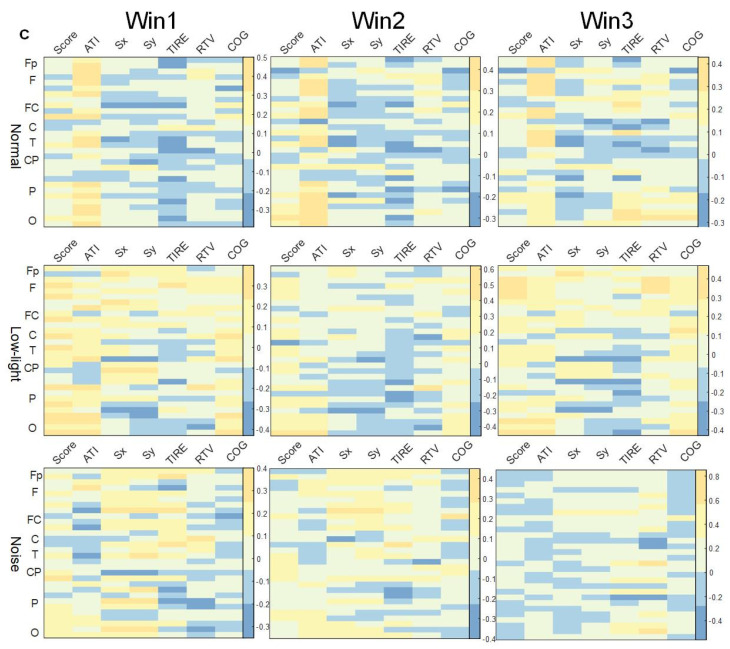
Correlation between ERD/ERS characteristics and shooting performance. (**a**) Theta band correlation; (**b**) alpha band correlation; (**c**) beta band correlation. The figure shows the correlation difference between the ERD/ERS characteristics of the normal, low-light, and noise conditions and the corresponding shooting performance in different time windows of the theta, alpha, and beta bands (Win1, Win2, and Win3), where, (**a**) represents the theta frequency band; (**b**) stands for the alpha band; (**c**) stands for the beta band. The data in the figure are Pearson correlation coefficients. The vertical axis represents the leads of the corresponding brain regions. The color difference in the figure represents the size of the correlation coefficient. The colder the color, the smaller the correlation coefficient, and the warmer the color, the larger the correlation coefficient.

As shown in Figure 7a, there was no concentrated lead correlation between ERD/ERS characteristics in the theta band and the corresponding SCORE index in the normal, low-light, and noise conditions, and the correlation was weak. Among them, the normal condition showed a significant positive correlation in the prefrontal (Fp1) lobe (*p* < 0.05). In the low-light condition, there was a significant positive correlation between the right central (FC6), left central (C3, CP5), right parietal (P4), and right temporal (T8) lobes (*p* < 0.05). The noise condition showed a significant positive correlation in the right central region (FC6, CP2) (*p* < 0.05). In terms of the correlation between ERD/ERS characteristics and the corresponding ATI index, the low-light condition, noise condition, and normal condition were all significantly positively correlated (*p* < 0.05), the leads showing significant positive correlations were the same, and the leads showing significant correlations were distributed in the prefrontal, frontal, temporal, parietal, and occipital lobes. However, the correlation of the noise condition to this index gradually decreased with an increase in the time window, and the other two conditions did not change significantly with the time window. The correlation between the low-light condition, the noise condition, and the normal condition was different in the Sx index. The ERD/ERS characteristics of the noise condition were positively correlated with the Sx index, and the positive correlation was strong. However, for the Sy index, the normal condition and the low-light condition had a strong negative correlation with the frontal and parietal regions which was not reflected in the low-light condition. In the correlation of the RTV index, the positive correlation of the noise and normal conditions became stronger with an increase in the time window, while the correlation of the low-light condition was weak and did not change significantly with the change in the time window.

#### 3.5.2. Correlation between Shooting Performance and ERD/ERS Characteristics in Alpha Band

As shown in Figure 7b, the correlation analysis of the SCORE index showed that the ERD/ERS characteristics in the normal condition had no lead significantly correlated with this index in the three time windows. In the low-light condition, significant positive correlations were found in the left central (FC1), right central (FC6, CP2, CP6) and left temporal (T7) regions (*p* < 0.05). In the noise condition, the left central (FC5) and central (Cz) regions showed a significant negative correlation (*p* < 0.05). The correlations between ERD/ERS characteristics and ATI indicators were also different in each condition, and the normal condition wase significantly positively correlated with this index in Fp1, Fp2, Fz, F4, F8, FC6, T7, T8, P7, P8, PO4, Oz, O1, and O2 (*p* < 0.05). In the low-light condition, more leads showed significant correlations in Win2 than in Win1 and Win3, and the significant nodes (*p* < 0.05) were Fp1, Fp2, F3, F4, F7, F8, FC1, FC2, FC6, C3, T8, CP1, CP2, P8, and O2, and their significance was a significantly positive correlation. The noise conditions did not show significant correlations in Win3, and the strongest significant positive correlations were in Win2. The significant nodes were Fp1, Fp2, F4, F8, FC2, FC6, Cz, and PO4, with the most prominent significant positive correlations at prefrontal Fp1 (r = 0.42 *p* < 0.05) and Fp2 (r = 0.62 *p* < 0.01).

#### 3.5.3. Correlation between Shooting Performance and ERD/ERS Characteristics in Beta Band

As shown in Figure 7c, in the beta band, the correlation under the noise condition was significantly weaker compared to both normal and low-light conditions. Additionally, the number of significantly correlated nodes is fewer than in the other two conditions. In the correlation between ERD/ERS characteristics and the corresponding SCORE index in the beta band, the correlation was stronger in the low-light condition than in the normal condition and the noise condition, and the significant positive correlation was also stronger than in the other two conditions, and the leads showing a significant positive correlation (*p* < 0.05) were F3, F7, P3, P8, PO4, and O2; the normal condition showed a significant positive correlation at FC6 in Win1 and at O1 in Win2. The normal conditions showed a significant positive correlation (*p* < 0.05) at FC6 of Win1 and at O1 of Win2, and no significant correlation node was found in Win3; no significant correlation node was found in the noise condition. Regarding the correlations of the ATI indexes, the normal condition showed similar levels of significance in Win1 and Win2, but the difference was not significant, and significant positive correlation (*p* < 0.05) nodes were concentrated in the prefrontal (Fp1, Fp2), frontal midline (Fz), frontal (F3, F4, F8, FC6), temporal (T7, T8), and parietal (P4, P8, PO3, PO4) areas. In contrast to Win1 and Win2, no significant correlations were found in the parietal and occipital lobes in Win3; in the low-light conditions, no significant correlations were found in the frontal lobes, and the nodes with significant positive correlations (*p* < 0.05) were P3, PO4, Oz and O2; in the noise conditions, significant positive correlations were found only at the F3 node in the beta band in Win1.

## 4. Discussion

The purpose of this study is to compare the differences in EEG band power characteristics, ERD/ERS characteristics, and the correlation between EEG band power characteristics and ERD/ERS characteristics in different environments and shooting behavior indicators of skilled shooters in normal and visual–auditory-limited environments in order to investigate changes in the active regions of a shooter’s brain in the phase of shooting and aiming in the context of interference due to noise and low light.

### 4.1. Difference Analysis of Shooting Behavior Indicators

As a sport requiring a high level of concentration, the shooting performance of shooters is closely related to environmental conditions. The athletic performance of shooters in shooting is easily affected by external conditions, and the shooting performance of shooters in different conditions may have a very significant difference [23]. This study evaluated the shooting performance of skilled shooters in three different environments. It was found that the different shooting environments caused the shooters to perform differently than they would in a normal environment under visual and auditory limitations.

The smaller the values of Sx and Sy, the stronger the gun stability. As can be seen from Figure 2, a low-light environment greatly affects the gun stability of shooters, while noise also has an impact, but the difference between the two indexes and the normal condition is not obvious. The COG and ATI indexes represent a shooter’s aiming ability. Both low-light and noisy environments have significant influence on this index, and the index values are smaller than those for the normal condition. However, the influence of the low-light environment on the shooter is obviously stronger than that of the noisy environment. The RTV and TIRE indexes represent the control ability of the shooter to pull the trigger when shooting. The smaller the RTV value is, the easier the trigger is to pull. From the performance of this index, low light has a greater impact on a shooter. Low light has a positive effect on the quality of the shooter’s trigger, and noise has a negative effect. The best representation of the shooters’ shooting performance was the SCORE; the low-light condition was the worst, and the noise conditions and the normal conditions resulted in no significant difference. Overall, low light had the strongest effect on the shooters’ performance, followed by noise.

### 4.2. Difference Analysis of Relative EEG Frequency Band Power

The present study examined the differences in the relative EEG band power in the brains of skilled shooters under three conditions. Previous studies suggested that the theta band is usually involved in memory encoding and retrieval, working memory, etc. [24,25,26]. The alpha band is associated with attention, conscious thinking and integration, plays an important role in the integration of brain structures in sensory and cognitive activities, and is mainly found in brain regions not involved in work [27]. The beta band mainly occurs when people are in states of attentiveness, active thinking, and alertness [28]. We examined significant differences in the relative EEG band power of the three conditions in different frequency bands. On the whole, there were fewer nodes with significant differences in the alpha band, indicating that the shooters’ attention and thinking integration levels were less affected by audiovisual restrictions.

The EEG relative band power in the theta band is stronger in the central versus frontal lobes, and the power in the frontal lobes is stronger in a noisy environment than in a control environment; previous studies found that prolonged audition stimulation significantly increases power in the frontal and central regions in the theta and alpha bands and that total power increases in the right central region [29]. The frontal lobe is responsible for immediate and sustained attention, etc., while the central region is the sensory and motor cortex [30]. It can be seen that in a noisy environment compared to a control environment, a shooter’s frontal lobe will be more active with stronger arousal when shooting during noise disturbance in order for the shooter to be able to focus and overcome the noise disturbance in order to achieve the level of shooting exhibited during normal training.

The intensity of EEG relative band power in the alpha band is manifested in the parietal lobe and the occipital lobe, which comprise the center of the visual cortex [31]; the parietal lobe is responsible for solving mathematical processing, spatial recognition, etc., through which the human brain plays a dominant role in processing short-term spatial memory information [32]. In the low-light environment, the shooters’ EEG relative band power intensity in the occipital and parietal lobes gradually increased with the time window and was stronger compared to the control and low-light conditions, indicating that the low-light environment made the shooters’ visual cortical centers and processing spatial information functions more active to better adapt to the darkness and accomplish higher levels of shooting.

The presence of beta-band signals is associated with excitement and strong emotions [33]. In the low-light condition, the beta band demonstrated strong EEG power in the right and left temporal regions, the right hemisphere of the frontal lobe, which is responsible for immediate and sustained attention, and the temporal lobe, which has an auditory center; additionally, according to previous studies, the temporal lobe is closely linked to functional areas of the brain while a shooter aims [6,8,34]. In the low-light environment, the frontal and temporal lobes were more active in the skilled shooters compared to the normal and low-light conditions, which enabled the shooters to maintain better attention; additionally, the temporal lobes were stronger, which enabled the shooters to maintain better concentration due to the suppression of auditory perception.

### 4.3. Difference Analysis of ERD/ERS

In this study, ERD/ERS features were extracted from the shooting preparation phase of skilled shooters under audiovisual limitations and in a normal environment to analyze the neural mechanisms of self-regulation in the brains of skilled shooters in the face of noisy and low-light environments. In the theta band, all three conditions had an ERD amplitude in the central frontal region except for the normal condition, for which the ERD amplitude was the strongest in Win2 and weakened in Win3, and the ERD amplitude in the central frontal region in the low-light and noisy conditions, for which the ERD amplitude increased as the shooting point amplitude was approached. Previous research has suggested that ERD is associated with higher cognitive processes such as working memory and reflects present attention [35,36]. When people perform a certain task, such as shooting task, it is inevitable to encounter interference from surrounding environmental factors (noise, low light). In order to quickly adapt to interference, the brain will inhibit the higher cognitive functions located in the frontal lobe [37]. Therefore, in order to adapt to the interference caused by noisy and low-light environments, the brain of a skilled shooter actively inhibits the higher cognitive functions in the frontal area, and the inhibition of this function gradually increases as the shooting point approaches. In this frequency band, ERS characteristics were observed in the right central region in the low-light environment, but the amplitude of ERD in the right central region gradually increased with the time window in the noisy environment. The activation of the right central zone awakens calm emotions [30]. In low light, the function of the shooter’s right central region is inhibited, while in a noisy environment, the opposite is observed. It can be seen that in a noisy environment, the brain has difficulty regulating more complex calm emotions, but in low light, this brain region is gradually active. This finding suggests that low light makes skilled shooters smoother than in normal conditions, but noise inhibits this function. The reason for which shooting performance in the noise condition was not as good as in the other two conditions was provided.

Previous studies have shown that alpha bands are one of the physiological mechanisms underlying neural efficiency in the preparation of visuomotor performance, reflecting functional patterns of thalamocortical and corticocortical circuits that facilitate or inhibit the transmission and retrieval of sensorimotor and cognitive information to the brain [38]. In addition, it has been found that trained motor tasks are performed with the inhibition of cognitive processes [34]. In the low-light condition, the shooters showed strong ERD characteristics in the central lobe, and its amplitude increased with the approach of the shooting point. Moreover, the central lobe was associated with physical cognitive ability, suggesting that the shooters’ cognitive ability for shooting tasks was inhibited in this environment, which may involve task memory. It has been found that the left sensorimotor cortex (C3) may enhance logical ability on cognitive tasks [39]. In the low-light condition, the ERS signature was found at alpha band C3, suggesting that the low-light condition enhanced the brain’s logical ability for cognitive tasks, similar to the normal condition but not the noise condition. Therefore, the a shooter’s logical ability for cognitive tasks is reduced in noisy environments compared to normal environments.

Beta-ERD reflects cortical activation, and beta-ERS reflects cortical deactivation; thus, less beta-ERD or more beta-ERS leads to higher beta synchrony and a greater inhibitory effect on motor activity [40]. In the beta band, shooters in the normal condition showed strong ERS characteristics in the central parietal region, and in the noise environment, shooters also showed ERS characteristics in the central parietal lobe, but the amplitude was smaller than in the normal environment, while in the low-light condition, shooters did not show ERS characteristics. The parietal lobe plays a dominant role in processing short-term spatial memory information [41]. The results indicate that the processing of spatial memory information was inhibited when shooting in a low-light environment. Although the central parietal lobe was also activated in the noisy environment, the degree of activation was much lower than in the normal environment. Moreover, spatial recognition ability was stronger when the shooter was near the shooting point in the noisy environment. In the low-light environment, the amplitude of ERD in the shooter’s central region was enhanced with an increase in the time window. The situations in the central region in the normal environment and noisy environment were similar, both of which were ERS. The low-light environment inhibited the motor sensory center of the shooters, while noisy environment and normal environments had no significant effect on this function, and this function was activated.

### 4.4. Correlation Difference Analysis of Shooting Performance and EEG Power Characteristics

The present study examined the correlation between EEG relative band power and shooting performance for all bands in different environments using a correlation analysis. It was found that EEG power characteristics in the theta band correlated more intimately with shooter’s shooting performance than the other two bands. Previous studies have found that the frontal cortex is primarily responsible for high-level task planning and attention; parietal areas are primarily responsible for sensory–motor information processing [42]. EEG power was significantly correlated with gun stability in the noise condition only at nodes Fz, FC2, and P8 in Win1. The significant correlation between EEG power and gun stability in the normal condition and the low-light condition was concentrated in the frontal lobe, left and right temporal regions, central region, and parietal lobe, indicating that in this frequency band, noise greatly affects the correlation between the gun stability index and the corresponding EEG power. The right temporal region is responsible for visuospatial tasks [43]. Shooting performance is the index that can directly reflect the shooter’s shooting level. In the low-light environment, a significant negative correlation was found in the central region, the left and right temporal regions, and the parietal lobe, indicating that the shooter’s attention to advanced tasks and the ability to process visuospatial tasks have a negative impact on the shooter’s performance when vision is limited. Trigger control ability was also used as an indicator of shooting quality. In the low-light environment, the EEG power features of the frontal, frontal, central, and parietal lobes showed a significant positive correlation with this indicator, while the significant correlation between the noise condition and the normal condition was a significant negative correlation, and the significant node was not concentrated in one brain region. These results indicate that the low-light environment has a positive effect on the function of each brain region and the trigger control ability of the shooter, while the effects of the normal environment and the noisy environment are not obvious.

Similar to the alpha and beta bands, the correlations between EEG power and various indicators in the noisy environment were not significantly different from those in the normal environment. The correlation distribution is basically the same, but the low-light environment is significantly different from the normal environment. Firstly, the low-light environment has significant negative correlations with corresponding performance indicators in the frontal region, central region, and parietal lobe. Secondly, gun stability was similar to that in theta band, and significant positive correlations were concentrated in the frontal region, left and right temporal region, central region, and parietal lobe. Second, the correlation of trigger control ability was also similar to that of the theta band, with significant positive correlation lines concentrated in the prefrontal region, frontal region, central lobe, and parietal lobe. It can be seen that the frontal lobe, which is responsible for advanced information processing, the central lobe of the sensory and motor cortex, the parietal lobe, which is responsible for sensorimotor function, and the occipital lobe, which is responsible for visual perception and visuospatial motor functions [26,44,45], are involved in the process of coordinating shooting and aiming. Under the influence of a low-light environment, the brain’s ability to process higher-level information, a sensorimotor correlation, and visual sensorimotor function have positive effects on a shooter’s trigger control.

### 4.5. Correlation Difference Analysis of Shooting Performance and ERD/ERS Characteristics

A correlation analysis was also used to examine the correlation between ERD/ERS characteristics and shooting performance for all frequency bands in different environments. It was found that the ERD/ERS characteristics of the theta band were more closely correlated with the shooters’ shooting performance than the other two bands. In the theta band in the normal condition, the ERD/ERS characteristics were only found in the left prefrontal lobe (Fp1) in Win3, while the noise condition was found to have a significant negative correlation with the right central region in Win2 (FC6, CP2). The low-light condition showed more nodes with significant positive correlations than the other two conditions. They are concentrated in the frontal and central regions. Activity in the right central region evokes a peaceful mood [38]. In conclusion, there is a positive correlation between a shooter’s performance and the right central region of the brain when the shooter is disturbed by noise, and its performance is directly related to the shooter’s emotions when shooting. This conclusion also verifies the view that shooting preparation is a delicate mental activity [1]. Secondly, in the correlation discussion regarding the aiming ability index, the noise condition exhibits a stronger significant positive correlation. However, in Win3, the significant nodes are reduced, and they are not concentrated. The three conditions showed a significant positive correlation with aiming ability in the prefrontal, frontal, central, parietal, and occipital lobes, but the correlation in the low-light condition was weaker than that in the other two conditions, indicating that the low-light environment affected the shooters’ aiming ability, which reduced the positive correlation between the visual nerve center of the brain and aiming ability.

## 5. Limitations

In this study, we analyzed correlations between behavioral performance indicators, EEG characteristics, ERD/ERS characteristics and behavioral indicators and EEG characteristics, and ERD/ERS characteristics in skilled shooters in a normal environment, a low-light environment, and in the context of noise disturbance so that we could have some understanding of the differences in brain mechanisms in skilled shooters while shooting with audiovisual limitations; however, there are still some limitations of this study. Because the subjects of this paper were selected amongst skilled shooters, the brain mechanisms of top shooters in the same environment were not studied; additionally, while a considerable amount of subjects’ data was collected in this study, the amount of EEG data, etc., remained small, which led us to a very difficult interpretation of the correlation analysis and its differences, and very limited conclusions were drawn, such as the present conclusions on the relative band power characteristics of EEG and behavioral indicators in some leads. The simultaneous occurrence of significant positive/negative correlations was not analyzed for a reasonable scientific explanation. At the same time, the statistical analysis methods used for the data samples in this study were relatively monotonous and inflexible. In the next step, it is necessary to explore more statistical analysis methods and apply them flexibly to this area of research, such as permutation tests and *t*-tests. Again, since the acquisition of EEG data during the shooting–aiming phase requires the subject to complete the shooting behavior, such as pulling the trigger, the experimental paradigm is different from that of experiments that do not require physical actions such as motor imagery, so strong physiological signal artifacts will inevitably be generated, and the method used in this study to process the raw data is relatively simple, and the removal of interference signals is not ideal. Identifying means of better preprocessing EEG signals and performing a physiological analysis of the correlation between shooting indicators and EEG features is a future direction for exploring the differences in the neural mechanisms of the brain during shooting and aiming in different sensory-function-limited environments. Additionally, Domingos et al. noted in 2023 that despite advancements in EEG engineering, results obtained using wireless equipment should be viewed with care [46].

## 6. Conclusions

In summary, the present study analyzed the differences between EEG power characteristics, ERD/ERS characteristics, and shooting behavior indicators and the correlation with shooting performance in 40 skilled shooters during the shooting preparation phase at night in low-light, noise-interference, and normal environments. The results showed that the low-light condition had a greater effect on shooting performance. Shooting performance, gun stability, aiming ability, and trigger control were all affected more in the low-light condition than in the noisy condition. In terms of the relative EEG band power, the low-light condition was significantly different from the other two conditions in the beta band, and the power values of the prefrontal lobe and the left and right temporal regions were significantly stronger. The same difference was found in the alpha band, but the power intensity in the alpha band was concentrated in the parietal and occipital lobes, and the EEG power increased as time approached the shooting point. However, in the theta band, the midline and the midline of the forehead in the normal condition were stronger than those in the low-light and noise conditions.

## Figures and Tables

**Table 1:** Statistical data analysis and shooting performance results in low-light, noisy, and normal environments.

	Noise M (SD)	Low-Light M (SD)	Normal M (SD)	F	△ _(Normal–Noise)_	▲ _(Normal–Low-light)_
SCORE ***	8.50 (0.38)	7.71 (0.43)	8.55 (0.46)	85.73	0.05	0.84 b
ATI ***	3.14 (0.99)	2.76 (1.00)	3.75 (1.29)	14.86	0.61 a	0.99 b
SX ***	0.82 (0.23)	0.96 (0.29)	0.77 (0.22)	14.62	−0.05	−0.19 b
SY ***	0.96 (0.26)	1.19 (0.36)	0.94 (0.24)	16.76	−0.02	−0.25 b
COG ***	9.22 (0.49)	7.91 (0.65)	9.37 (0.57)	127.9	0.15	1.46 b
TIRE ***	2.00 (0.17)	2.12 (0.21)	2.03 (0.19)	6.900	0.03	−0.09 b
RTV	1.11 (0.47)	1.14 (0.61)	1.10 (1.91)	1.920	−0.01	−0.04

*** = *p* < 0.01; a = noise different from normal (post hoc); b = low light different from normal (post hoc).

**Table 2:** Significant differences among condition factors and time window factors in the theta band for EEG Relative Power—a two-way repeated measures ANOVA (Nor: normal condition; No: noise condition; L: low-light condition).

	Conditions							Time Window				
	M (SD)							M (SD)				
	F	Nor	L	No	Post Hoc			F	Win1	Win2	Win3	Post-Hoc
Fp1	4.58 **	0.48 (0.16)	0.45 (0.18)	0.52 (0.16)	No > L, Nor		Fp1	6.05 ***	0.52 (0.18)	0.44 (0.16)	0.50 (0.16)	1, 3 > 2
Fp2	7.07 ***	0.18 (0.17)	0.43 (0.20)	0.52 (0.17)	No > L, Nor		Fp2	5.94 ***	0.50 (0.19)	0.43 (0.17)	0.50 (0.18)	1, 3 > 2
F4	3.31 **	0.45 (0.13)	0.41 (0.17)	0.46 (0.13)	No > L, Nor		F3	3.52 **	0.51 (0.11)	0.48 (0.10)	0.49 (0.11)	1 > 2
F8	3.91 **	0.49 (0.17)	0.43 (0.19)	0.49 (0.17)	Nor, No > L		F7	4.47 **	0.55 (0.14)	0.50 (0.14)	0.52 (0.14)	1 > 2
T7	3.31 **	0.35 (0.14)	0.31 (0.13)	0.34 (0.12)	No, Nor > L							
CP2	4.29 **	0.56 (0.11)	0.54 (0.11)	0.52 (0.11)	Nor > No, L							
P7	4.58 **	0.38 (0.12)	0.34 (0.12)	0.38 (0.12)	Nor > L, No > L							
PO3	3.07 **	0.45 (0.12)	0.41 (0.12)	0.44 (0.13)	-							

** = *p* < 0.05; *** = *p* < 0.01.

**Table 3:** Significant differences among condition factors and time window factors in the alpha band for EEG relative power—two-way repeated measures ANOVA (Nor: normal condition; No: noise condition; L: low-light condition).

	Conditions							Time Window				
	M (SD)							M (SD)				
	F	Nor	L	No	Post Hoc			F	Win1	Win2	Win3	Post-Hoc
Fp1	6.12 ***	0.21 (0.05)	0.19 (0.06)	0.21 (0.06)	Nor > L, No > L		Fp1	7.96 ***	0.19 (0.05)	0.21 (0.06)	0.21 (0.06)	1 < 3, 2
Fp2	10.9 ***	0.20 (0.05)	0.17 (0.06)	0.20 (0.05)	Nor > L, No > L		Fp2	6.78 ***	0.18 (0.05)	0.20 (0.06)	0.19 (0.05)	1 < 2
F4	11.6 ***	0.24 (0.05)	0.21 (0.06)	0.21 (0.06)	Nor > L, No > L		Fz	3.35 **	0.24 (0.05)	0.26 (0.05)	0.26 (0.06)	-
F8	8.16 **	0.23 (0.07)	0.20 (0.07)	0.24 (0.08)	Nor > L, No > L		F3	6.55 ***	0.24 (0.05)	0.27 (0.06)	0.27 (0.06)	1 < 2, 3
T8	3.03 **	0.24 (0.08)	0.22 (0.08)	0.25 (0.07)	-		F4	3.14 **	0.22 (0.05)	0.24 (0.06)	0.24 (0.06)	-
P8	3.99 **	0.29 (0.05)	0.28 (0.07)	0.30 (0.05)	No > L, Nor		F7	4.73 ***	0.23 (0.08)	0.26 (0.09)	0.26 (0.09)	1 < 2
							F8	3.47 **	0.21 (0.08)	0.23 (0.08)	0.23 (0.07)	-
							FC5	3.32 **	0.26 (0.06)	0.28 (0.07)	0.28 (0.08)	-
							C4	3.14 **	0.26 (0.06)	0.27 (0.06)	0.28 (0.07)	-
							CP1	4.85 ***	0.26 (0.07)	0.27 (0.07)	0.29 (0.07)	1 < 3
							P4	3.15 **	0.29 (0.06)	0.30 (0.06)	0.31 (0.06)	-
							O2	3.41 **	0.30 (0.07)	0.31 (0.06)	0.32 (0.06)	1 < 3

** = *p* < 0.05; *** = *p* < 0.01.

**Table 4:** Significant differences among condition factors in the beta band EEG relative power—two-way repeated measures ANOVA (Nor: normal condition; No: noise condition; L: low-light condition).

Conditions					
M (SD)					
	F	Nor	L	No	Post-Hoc
Fp1	7.94 ***	0.30 (0.17)	0.35 (0.18)	0.27 (0.16)	L > No, Nor
Fp2	11.0 ***	0.32 (0.18)	0.39 (0.21)	0.28 (0.18)	L > Nor, L > No
Fz	5.71 ***	0.18 (0.07)	0.21 (0.10)	0.17 (0.08)	L > Nor, L > No
F3	3.08 **	0.24 (0.10)	0.27 (0.12)	0.23 (0.10)	-
F4	7.95 ***	0.31 (0.15)	0.38 (0.19)	0.30 (0.14)	L > Nor, L > No
F8	9.64 ***	0.28 (0.17)	0.36 (0.20)	0.27 (0.16)	L > Nor, L > No
FC6	4.46 **	0.31 (0.15)	0.37 (0.17)	0.31 (0.17)	L > Nor, No
T7	3.12 **	0.40 (0.17)	0.45 (0.17)	0.40 (0.16)	-
P7	5.04 ***	0.32 (0.13)	0.36 (0.15)	0.31 (0.13)	L > No, Nor
P8	5.09 ***	0.31 (0.12)	0.35 (0.14)	0.30 (0.12)	L > No, Nor

** = *p* < 0.05; *** = *p* < 0.01.

**Table 5:** Significant differences among condition factors and time window factors in the theta band for ERD/ERS—two-way repeated measures ANOVA (Nor: normal condition; No: noise condition; L: low-light condition). M indicates the mean values of ERD/ERS values, measured in percentage (%).

	Conditions						Time window				
	M (SD)						M (SD)				
	F	Nor	L	No	Post-Hoc		F	Win1	Win2	Win3	Post-Hoc
Fp1	9.98 ***	−9.9 (0.04)	−5.1 (0.07)	−14 (0.05)	L > Nor, No	Fp1	7.96 ***	−9.4 (0.03)	−15 (0.04)	−4.4 (0.06)	3 > 1 > 2
Fp2	8.81 ***	−8.7 (0.05)	−4.5 (0.08)	−13 (0.05)	L > No	Fp2	6.78 ***	−9.2 (0.03)	−15 (0.04)	−2.7 (0.08)	3 > 1 > 2
Fz	6.03 ***	−5.1 (0.04)	−2.5 (0.04)	−9.3 (0.03)	L > No	Fz	3.35 **	−6.1 (0.03)	−8.8 (0.03)	−1.9 (0.04)	3 > 2
F3	4.66 **	−7.0 (0.04)	−4.8 (0.04)	−10 (0.03)	L > No	F3	6.55 ***	−7.6 (0.02)	−11 (0.03)	−3.7 (0.04)	3 > 2
F4	4.74 ***	−8.1 (0.04)	−5.6 (0.04)	−12 (0.03)	L > No	F4	3.14 **	−7.9 (0.02)	−12 (0.03)	−4.9 (0.04)	3 > 2
F7	6.09 ***	−8.9 (0.03)	−5.7 (0.03)	−12 (0.04)	L > No	F7	4.73 ***	−7.2 (0.03)	−12 (0.04)	−6.8 (0.03)	3, 1 > 2
F8	4.13 **	−9.0 (0.03)	−7.3 (0.04)	−13 (0.03)	L > No	F8	3.47 **	−8.9 (0.02)	−13 (0.03)	−6.9 (0.03)	3 > 2
FC1	3.49 **	−4.4 (0.05)	−3.4 (0.05)	−9.3 (0.03)	L > No	FC1	3.32 **	−6.1 (0.03)	−10 (0.02)	−8.4 (0.04)	3 > 2
FC2	4.57 **	−5.3 (0.06)	−3.3 (0.05)	−11 (0.03)	L > No	FC2	3.14 **	−7.5 (0.03)	−11 (0.03)	−1.6 (0.07)	3 > 2
FC6	3.17 **	−6.6 (0.02)	−5.7 (0.03)	−9.8 (0.03)	-	FC5	4.85 ***	−5.9 (0.01)	−8.9 (0.02)	−2.2 (0.02)	3 > 2
CP1	4.09 **	−19 (0.09)	−1.3 (0.20)	3.53 (0.02)	No > L	FC6	3.15 **	−7.3 (0.03)	−10 (0.03)	−4.7 (0.02)	3 > 2
CP2	4.06 **	−2.1 (0.05)	0.43 (0.45)	−36 (0.18)	L > No	O1	3.41 **	−6.8 (0.01)	−10 (0.02)	−8.2 (0.03)	1 > 2
CP6	3.60 **	−3.4 (0.01)	−4.2 (0.01)	−8.2 (0.03)	-						
P4	3.52 **	−6.7 (0.03)	−6.9 (0.02)	−13 (0.03)	-						
P8	5.32 ***	−4.0 (0.02)	−3.9 (0.03)	−8.2 (0.02)	Nor, L > No						
PO3	3.32 **	−7.9 (0.02)	−7.9 (0.02)	−11 (0.02)	-						
PO4	4.84 ***	−8.0 (0.02)	−7.4 (0.02)	−12 (0.02)	L, Nor > No						
O1	4.06 **	−7.6 (0.02)	−7.2 (0.02)	−10 (0.02)	L > No						
O2	3.81 **	−8.4 (0.03)	−7.6 (0.03)	−11 (0.04)	L > No						

** = *p* < 0.05; *** = *p* < 0.01.

**Table 6:** Significant differences among condition factors and time window factors in the alpha band for ERD/ERS—two-way repeated measures ANOVA. (Nor: normal condition; No: noise condition; L: low-light condition). M represents the mean values of ERD/ERS values, measured in percentage (%).

	Conditions						Time Window				
	M (SD)						M (SD)				
	F	Nor	L	No	Post-Hoc		F	Win1	Win2	Win3	Post-Hoc
Fp1	7.60 ***	−7.2 (0.02)	−4.1 (0.03)	−10 (0.05)	L > No	Fp1	3.54 **	−7.1 (0.02)	−9.8 (0.03)	−5.0 (0.04)	3 > 2
Fp2	9.35 ***	−5.9 (0.03)	−3.5 (0.04)	−10 (0.03)	L, Nor > No	Fp2	5.84 ***	−6.4 (0.03)	−9.5 (0.04)	−3.8 (0.05)	3 > 2
Fz	8.25 ***	−0.1 (0.04)	5.01 (0.02)	−8.3 (0.04)	L > No	F3	3.06 **	−5.7 (0.03)	−6.2 (0.02)	0.16 (0.04)	-
FC1	5.34 ***	18 (0.06)	6.40 (0.04)	−3.2 (0.02)	Nor > No	F4	3.12 **	−4.4 (0.02)	−7.2 (0.02)	−2.0 (0.02)	3 > 2
Cz	5.28 ***	−53 (0.26)	−20 (0.04)	8.24 (0.03)	No > Nor	FC5	3.92 **	−4.8 (0.01)	−3.3 (0.01)	3.69 (0.01)	3 > 1
C3	3.31 **	12 (0.11)	13 (0.08)	−25 (0.04)	-	FC6	3.10 **	−4.2 (0.03)	−4.9 (0.02)	−0.03 (0.02)	-
T8	3.54 **	−3.5 (0.02)	−1.9 (0.02)	−5.7 (0.02)	L > No	T7	9.20 ***	−1.9 (0.01)	−0.3 (0.02)	4.59 (0.01)	3 > 2, 1
CP5	5.18 ***	23 (0.05)	−1.5 (0.04)	3.62 (0.09)	Nor > L	P3	4.20 **	−3.9 (0.05)	3.02 (0.04)	18 (0.01)	3 > 1
						P7	8.17 ***	−0.7 (0.01)	0.59 (0.01)	5.15 (0.01)	3 > 2, 1
						P8	3.86 **	−2.5 (0.01)	−2.6 (0.02)	1.43 (0.02)	-

** = *p* < 0.05; *** = *p* < 0.01.

**Table 7:** Significant differences among condition factors and time window factors in the beta band for ERD/ERS—two-way repeated measures ANOVA. (Nor: normal condition; No: noise condition; L: low-light condition). M represents the mean values of ERD/ERS values, measured in percentage (%).

	Conditions						Time Window				
	M (SD)						M (SD)				
	F	Nor	L	No	Post-Hoc		F	Win1	Win2	Win3	Post-Hoc
F4	3.59 **	−7.5 (0.04)	−3.3 (0.02)	−5.3 (0.03)	L > Nor	Fp1	5.01 ***	−0.4 (0.01)	−2.5 (0.01)	−4.3 (0.01)	1 > 3
F7	4.41 **	−5.7 (0.02)	−1.6 (0.01)	−4.4 (0.01)	L > Nor	Fp2	7.43 ***	−0.2 (0.01)	−3.2 (0.01)	−4.6 (0.01)	1 > 2, 3
FC5	5.76 ***	−4.4 (0.01)	1.29 (0.01)	−3.1 (0.01)	L > Nor, No	F4	5.54 ***	−2.1 (0.01)	−6.7 (0.03)	−7.4 (0.03)	1 > 2, 3
FC6	3.52 **	−5.8 (0.03)	−1.9 (0.01)	−2.2 (0.02)	L > Nor	P7	6.01 ***	1.33 (0.02)	2.02 (0.02)	6.92 (0.02)	3 > 2, 1
Cz	7.01 ***	21 (0.04)	−16 (0.07)	17 (0.08)	Nor, No > L	O1	3.65 **	−1.9 (0.01)	−3.4 (0.01)	2.02 (0.01)	3 > 2
C3	6.88 ***	−17 (0.09)	−8.7 (0.05)	19 (0.08)	L > No, Nor						
T7	6.78 ***	−3.5 (0.02)	−1.9 (0.02)	0.43 (0.00)	L, No > Nor						
T8	3.89 **	−2.4 (0.01)	−0.6 (0.01)	1.64 (0.01)	No > Nor						
CP5	4.22 **	5.01 (0.02)	27 (0.21)	−5.3 (0.06)	L > No						

** = *p* < 0.05; *** = *p* < 0.01.

## Data Availability

The data presented in this study are available on request from the corresponding author. The data are not publicly available due to [privacy concerns related to study participants].
